# Supplementary material for: p62 filaments capture and present ubiquitinated cargos for autophagy
Source: EMBO J. 2018 Jan 17;37(5):e98308. doi: 10.15252/embj.201798308 (PMC5830917; doi:10.15252/embj.201798308)
Supplement: Supplementary file 1 — Expanded View Figures PDF [file EMBJ-37-e98308-s001.pdf]

Expanded View Figures

Figure EV1. p62 and ubiquitin-positive proteins spontaneously cluster in solution.

A Representative micrographs of a cluster formation assay conducted with 2 μM mCherry-p62, 2 μM GFP-p62, and 5 μM GST-4xUb taken after 60 min from the reaction start.

B Schematic representation of the particle analysis workflow.

C Panel identical to Fig 1C, except for the fact that background was not subtracted from the pictures.

D Representative structured illumination microscopy (SIM) micrographs of p62 clusters formed with the indicated proteins. GFP-p62-containing clusters were formed for 30 min, and then, the respective mCherry-p62 mutants were added and incubated for another 30 min. Representative images of at least 10 particles per sample.

© 2018 The Authors

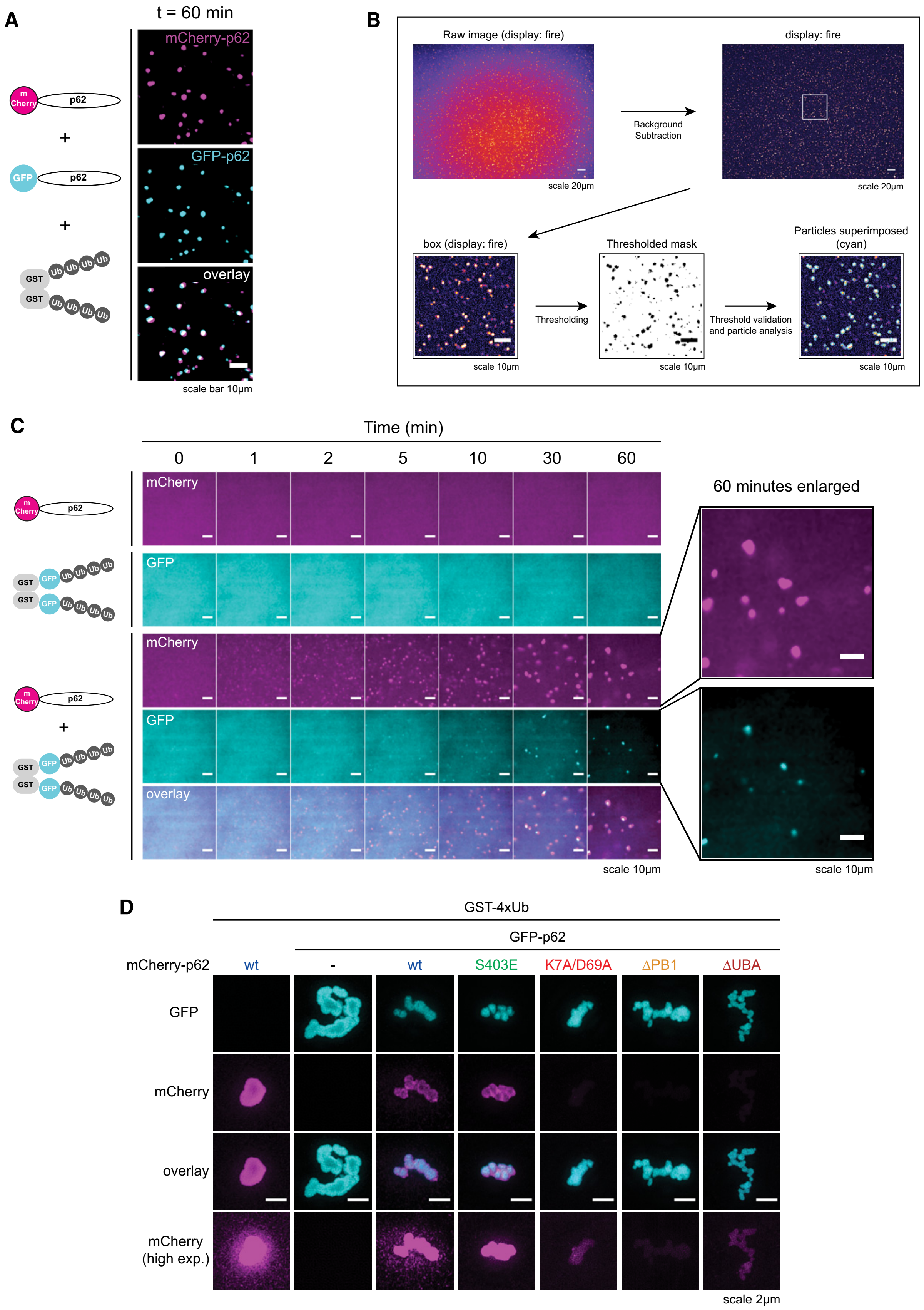
A
t = 60 min
mCherry-p62
GFP-p62
overlay
scale bar 10μm
p62
GFP
GST
Ub
B
Raw image (display: fire)
display: fire
Background Subtraction
scale 20μm
box (display: fire)
Thresholded mask
Particles superimposed (cyan)
Thresholding
Threshold validation and particle analysis
scale 10μm
C
Time (min)
0
1
2
5
10
30
60
mCherry
GFP
overlay
60 minutes enlarged
scale 10μm
D
GST-4xUb
GFP-p62
mCherry-p62
wt
-
S403E
K7A/D69A
ΔPB1
ΔUBA
mCherry (high exp.)
scale 2μm

Figure EV1.

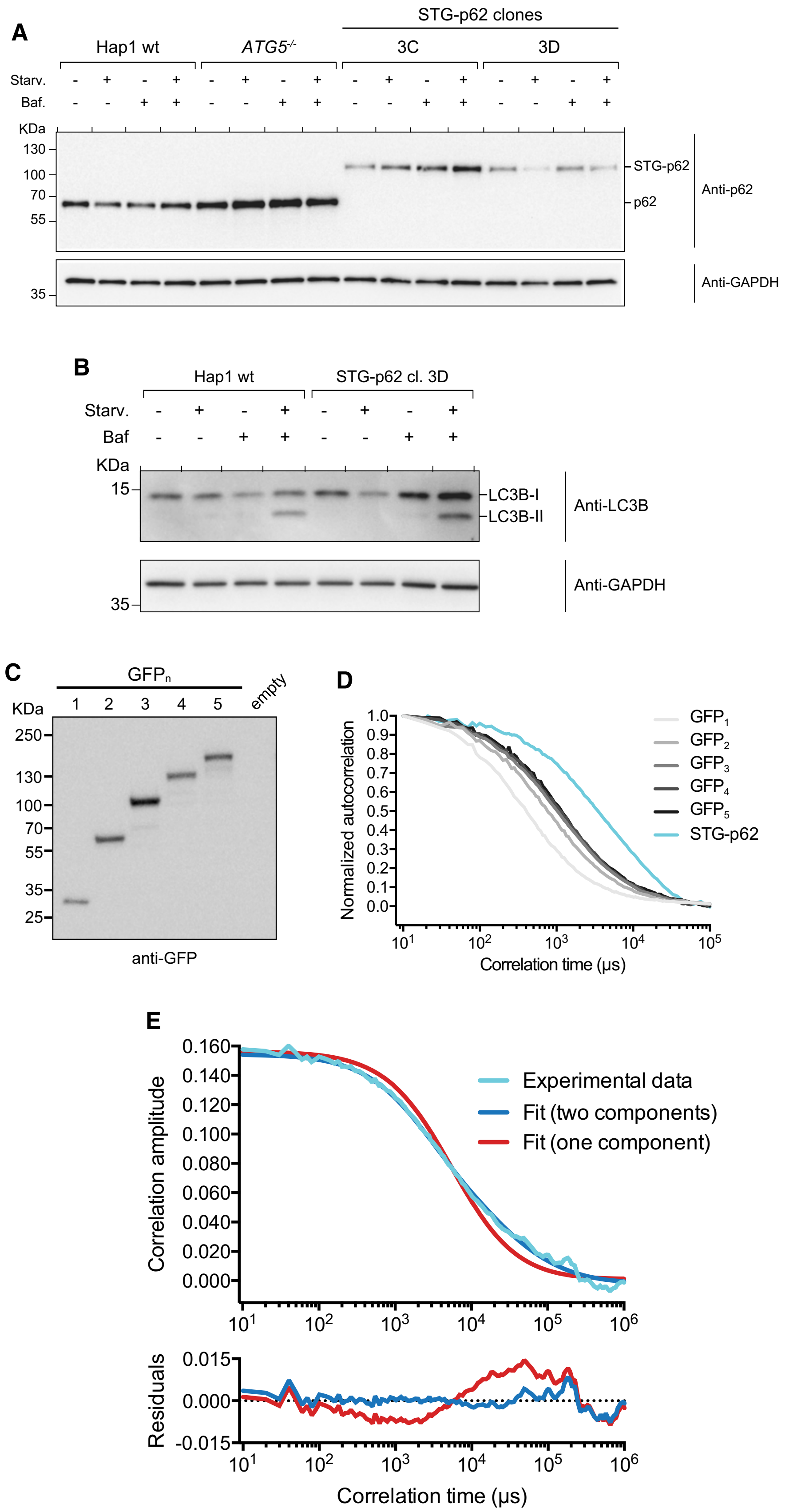

Figure EV2. Endogenously tagged p62 is oligomeric and forms clusters with ubiquitin.

A Characterization of the p62 degradation pattern upon starvation in two STG-p62 clones. Wild-type and *ATG5*$^{-/-}$ Hap1 cells were included as positive and negative controls, respectively. Baf.: bafilomycin A1. STG-p62 clone 3D was employed for further characterization and in the experiments shown in the main Figure. Anti-GAPDH blot is shown as loading control.

B Characterization of the LC3B lipidation pattern in wild-type and STG-p62 Hap1 cells upon starvation and/or bafilomycin A1 treatment. Anti-GAPDH blot is shown as loading control.

C Wild-type Hap1 cells transfected with the $GFP_{1\text{-}5}$ ruler constructs were lysed and subjected to Western blotting against GFP. A mock transfection was used as a control.

D Representative autocorrelation curves for the $GFP_{1\text{-}5}$ ruler constructs and STG-p62. Curves were normalized setting the first value to 1 and the last to 0. Three random curves per sample were averaged and plotted. See Materials and Methods for the detailed number of curves analyzed per sample.

E Top: a representative autocorrelation curve of STG-p62 (cyan) was fitted assuming either a single diffusing species (red) or two (blue). Bottom: plot of the residuals (data minus fit) for both models.

© 2018 The Authors

Figure EV3. p62-dependent cluster formation is triggered by poly-ubiquitinated proteins.

A Representative images of cluster formation assays conducted with 5 μM GST-4xUb and the indicated mCherry-p62 concentrations. Images were taken before the addition of GST-4xUb or 60 min after. Representative images of three independent replicates.

B Quantification of p62 cluster formation in the presence of the indicated free ubiquitin chains. GST-4xUb was used as a positive control. Averages and SDs from three independent replicates are shown. Samples were imaged for 15 min and then again after 45 min. Dashed lines represent the extrapolated curves for a full time lapse. For GST-4xUb data points from the peak of the curve to the latest time point (t = 45 min) were fitted to a single exponential decay (R^2 = 0.9973). Box: representative micrographs of the indicated samples 45 min after addition of Ub chains. Pictures are displayed in false colors (ImageJ: fire).

C Left: Coomassie Brilliant Blue-stained gel showing the assembly of GST-tagged K48- and K63-linked ubiquitin chains. Sup.: supernatant. Right: purified GST-tagged ubiquitin chains were subjected to Western blotting with the indicated antibodies.

D Left: quantification of aggregation assays conducted with 2 μM mCherry-p62 and 20 μM GST-4xUb in the presence or absence of 200 μM GFP-Ub, GFP, or Ub. Averages and SDs from four independent experiments are shown.

E mCherry-2xFKBP-p62 was subjected to size-exclusion chromatography in the presence or absence of the homodimerizer AP20187. Fractions were collected and run on a SDS–PAGE gel. The corresponding elution volume is indicated for each fraction.

F Cluster formation assays conducted with mCherry-p62 WT or -2xFKBP in the presence or absence of GST-4xUb and AP20187. Average and SDs of three independent experiments are shown.

© 2018 The Authors

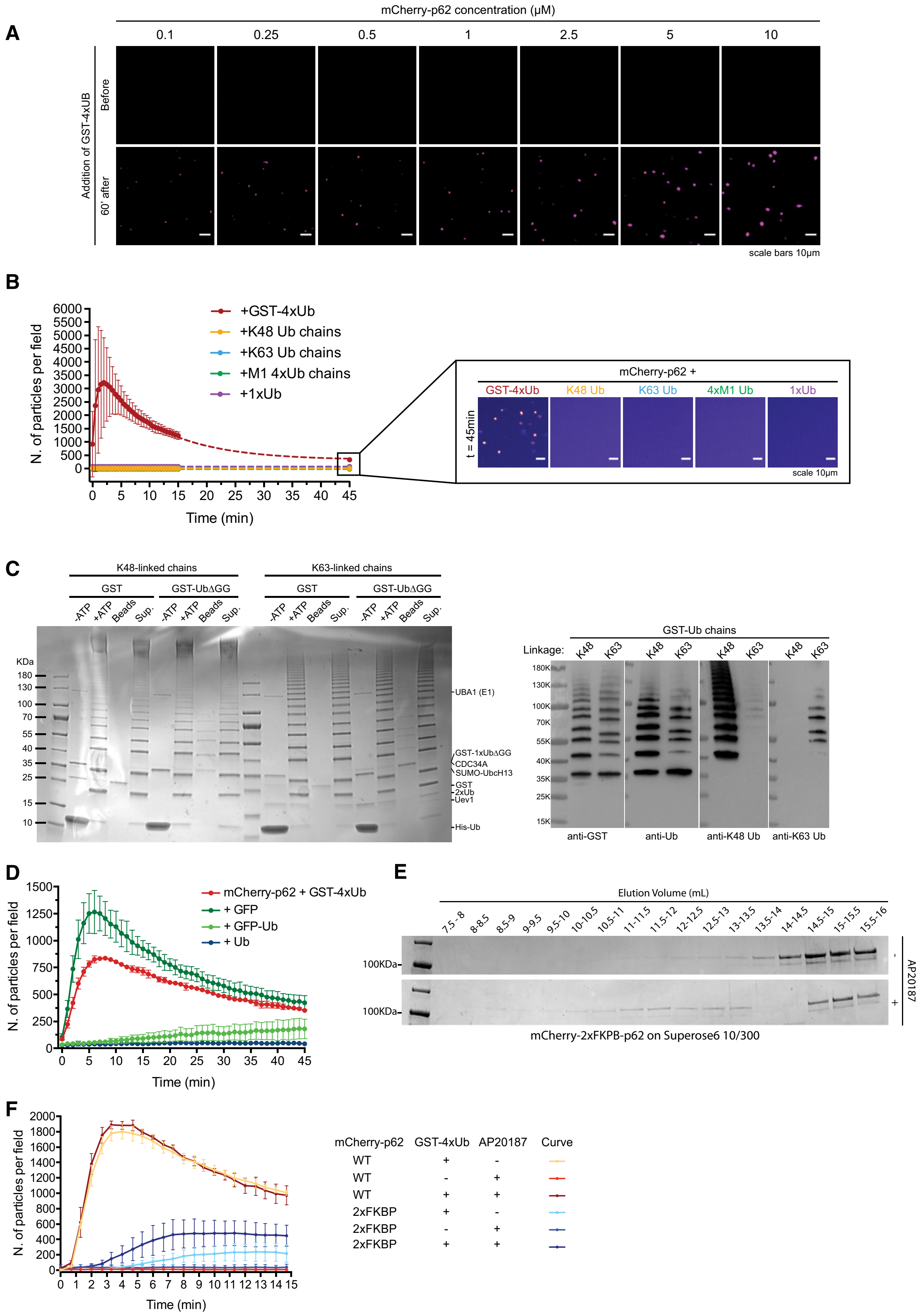

Figure EV3.

© 2018 The Authors

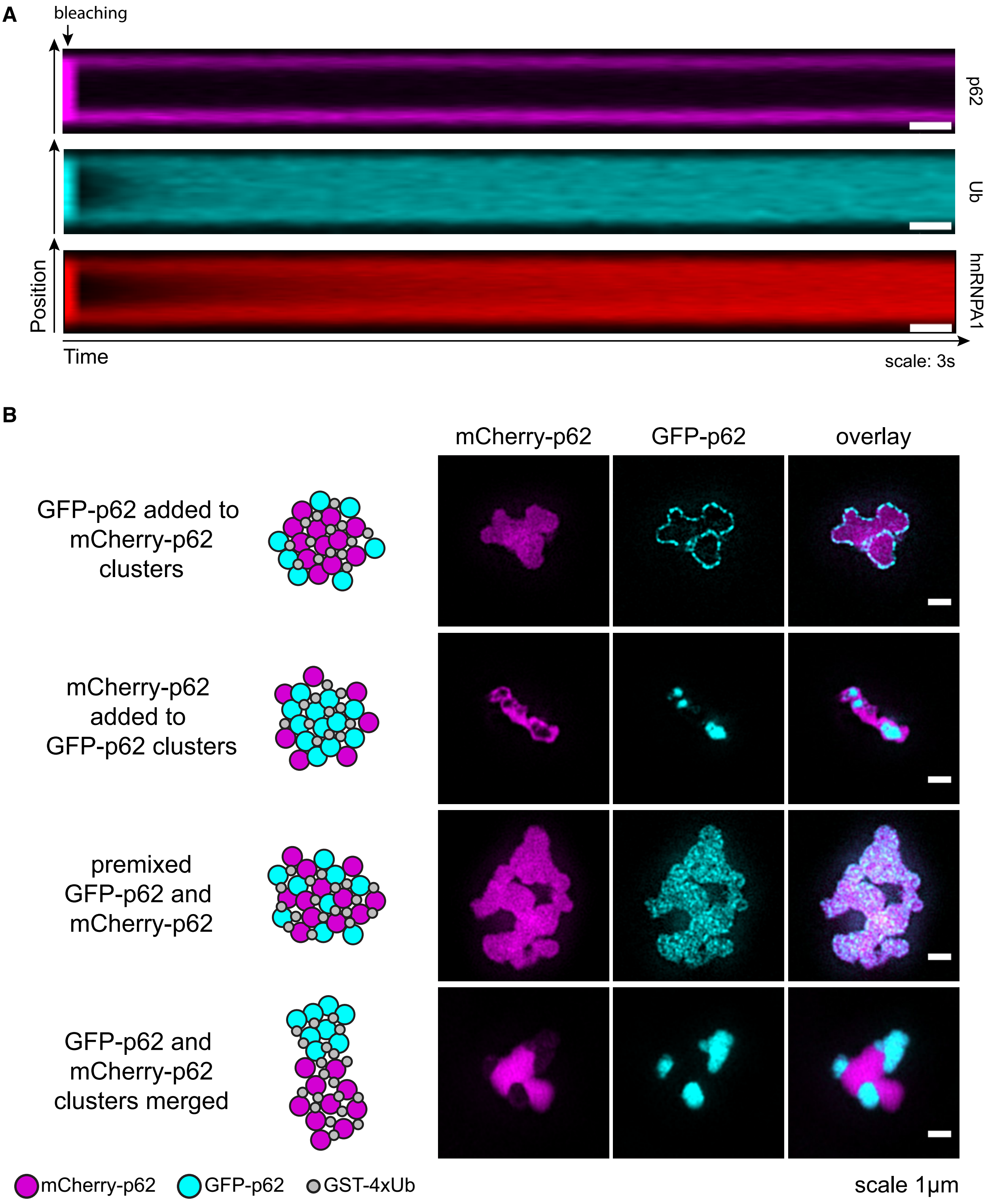

Figure EV4. p62 and ubiquitin show different motilities within the clusters.

A Representative kymographs of the samples shown in Fig 4A.

B Structured illumination imaging (SIM) was performed with GFP- and mCherry-p62-containing particles formed as indicated on the left. Representative micrographs of at least 20 particles per sample from two independent experiments.

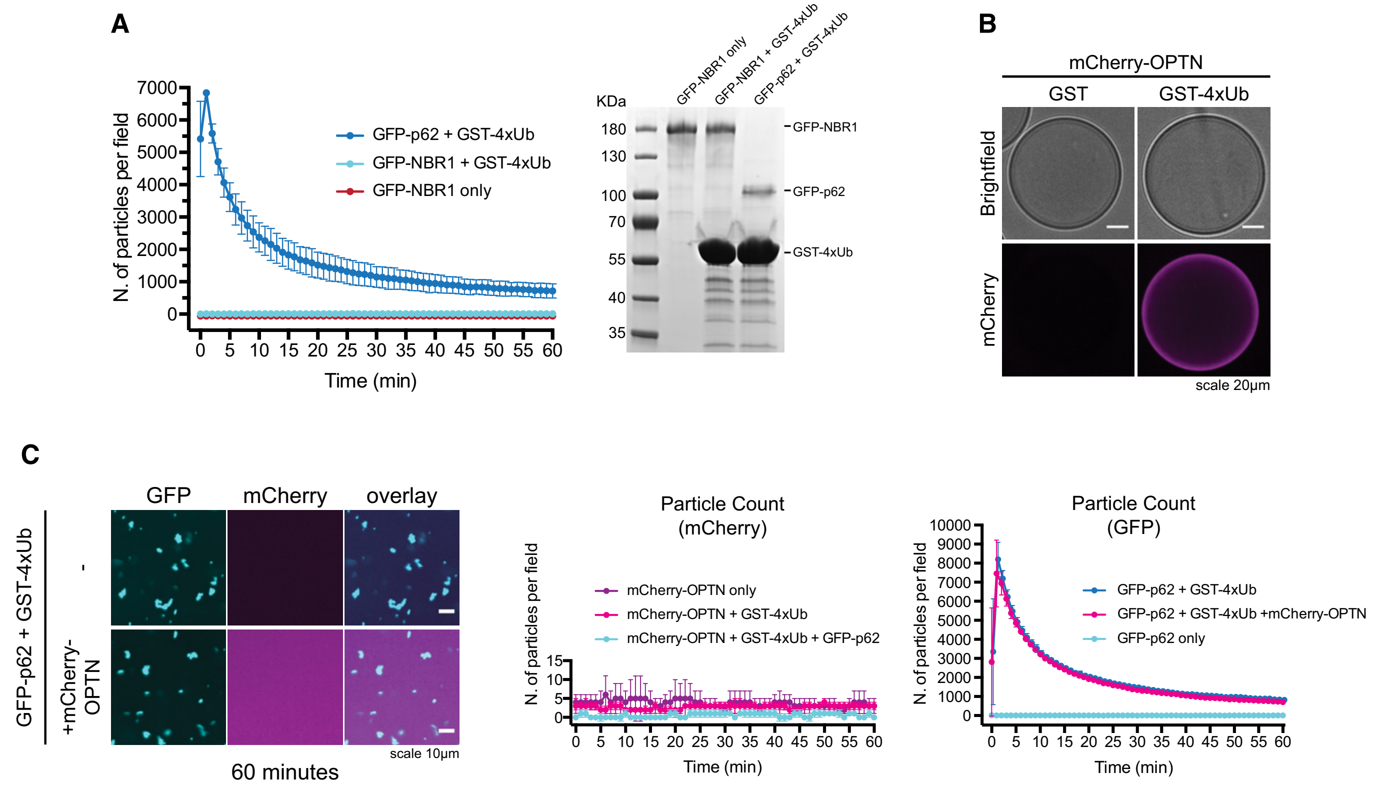

Figure EV5. NBR1 cooperates with p62.

A Left: quantification of cluster formation assays conducted with 2 µM GFP-p62 or GFP-NBR1 in the presence of 20 µM GST-4xUb. Averages and SDs from three independent replicates are shown. Right: Representative Coomassie-stained gel of the samples collected after imaging and run on an SDS–PAGE gel.

B mCherry-OPTN was recruited to GST- or GST-4xUb-coated beads and imaged by spinning disk microscopy. Representative images of at least 80 beads imaged per sample from three independent experiments.

C Left. Representative micrographs of cluster formation assays conducted with GFP-p62 and GST-4xUb in the presence or absence of mCherry-OPTN. The displayed pictures were taken 60 min after the addition of GST-4xUb. Middle and right: quantification of the number of particles formed by mCherry-OPTN (middle) and GFP-p62 (right) in the indicated samples. All graphs show averages and SDs from three independent experiments.

Figure EV6. LIR-mediated cross talk between cluster formation and the autophagy machinery.

A Representative micrographs of clusters formed with 1 µM mCherry-p62 WT or LIR mutant and 20 µM GST-4xUb for 10 min, followed by the addition of 1 µM GFP or GFP-LC3B immediately prior to imaging. Clusters deposited on the bottom of the plate were imaged by spinning disk microscopy at 63× magnification.

B GFP-p62- and GST-4xUb-containing clusters were formed in the presence of pre-mixed 20 µM mCherry or mCherry-LC3B employing p62 wild type or the LIR mutant. The maximal number of particles per field formed per sample was taken as readout. Data were normalized to the samples containing p62 and GST-4xUb only for both p62 wild type and the LIR mutant. Averages and SDs from three independent experiments are shown. *P*-values were calculated with a two-tailed unpaired Student's *t*-test.

C Quantification of cluster formation reactions conducted with 2 µM mCherry-2xFKBP-p62 in the presence of 5 µM GST-4xUb and 20 µM of the indicated proteins. Averages and SDs from three independent replicates are shown.

D Representative SIM micrographs of p62 clusters formed with the indicated proteins. GFP-p62-containing clusters were formed for 30 min, and then, the respective mCherry-p62 mutants were added and incubated for another 30 min. Representative images of at least 10 clusters per sample are shown.

E, F The indicated p62 variants were recruited to glutathione beads coated with the indicated GST-tagged proteins and imaged under a spinning disk microscope (E) or a conventional confocal microscope (F). Representative pictures of two independent experiments per condition are shown.

© 2018 The Authors

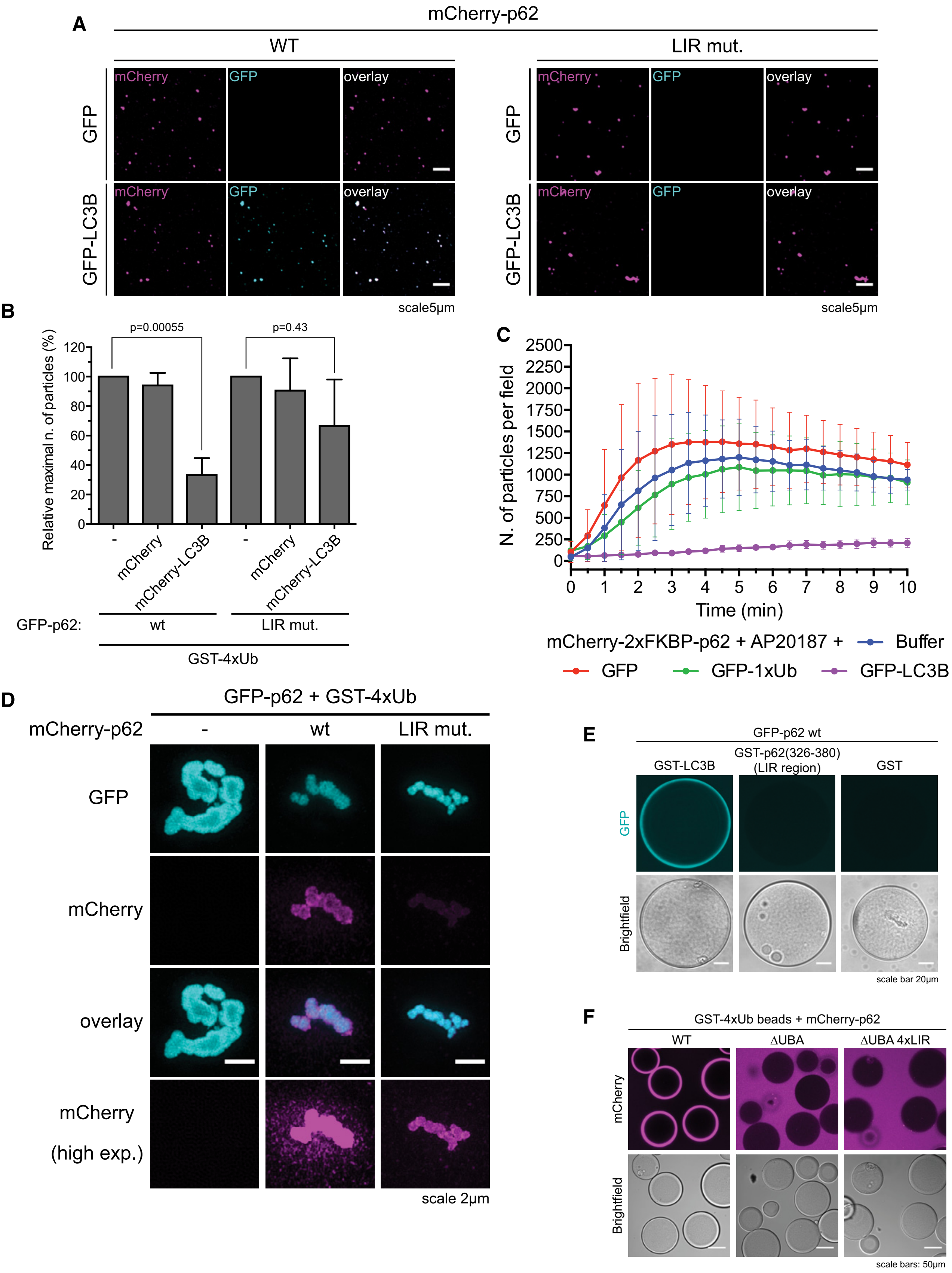
A
mCherry-p62
WT
LIR mut.
GFP
GFP-LC3B
mCherry
GFP
overlay
scale5μm
B
Relative maximal n. of particles (%)
p=0.00055
p=0.43
-
mCherry
mCherry-LC3B
GFP-p62:
wt
LIR mut.
GST-4xUb
C
N. of particles per field
Time (min)
mCherry-2xFKBP-p62 + AP20187 +
Buffer
GFP
GFP-1xUb
GFP-LC3B
D
GFP-p62 + GST-4xUb
mCherry-p62
-
wt
LIR mut.
GFP
mCherry
overlay
mCherry
(high exp.)
scale 2μm
E
GFP-p62 wt
GST-LC3B
GST-p62(326-380)
(LIR region)
GST
GFP
Brightfield
scale bar 20μm
F
GST-4xUb beads + mCherry-p62
WT
ΔUBA
ΔUBA 4xLIR
mCherry
Brightfield
scale bars: 50μm

Figure EV6.
