# Supplementary material for: p62 filaments capture and present ubiquitinated cargos for autophagy
Source: EMBO J. 2018 Jan 17;37(5):e98308. doi: 10.15252/embj.201798308 (PMC5830917; doi:10.15252/embj.201798308)
Supplement: Supplementary file 2 — Table EV1 [file EMBJ-37-e98308-s002.docx]

**Expanded View Table 1: Mass Spectrometry analysis of STG-p62 purified from Hap1 cells.**

|  | | Experiment 1 | | | Experiment 2 | | | Mean | |
| --- | --- | --- | --- | --- | --- | --- | --- | --- | --- |
| Gene Name | **Protein Name** | **Score** | **Sequence**  **coverage (%)** | **Log2(LFQ)**  **(STG-p62/wt)** | **Score** | **Sequence coverage (%)** | **Log2(LFQ)**  **(STG-p62/wt)** | **Log2(LFQ)_Avg_**  **(STG-p62/wt)** | **Fold enrichment (STG-p62/wt)** |
| SQSTM1 | p62/Sequestosome 1 | 130.0 | 24.9 | 12.0 | 323.3 | 65.7 | 13.0 | 12.5 | 5792x |
| KEAP1 | Kelch-like ECH-associated protein 1 | 28.9 | 4.6 | 4.4 | 103.1 | 27.6 | 3.0 | 3.7 | 13.0x |
| NBR1 | Next to BRCA1 gene 1 protein | 11.1 | 1.9 | 3.4 | 30.8 | 8.1 | 3.4 | 3.4 | 10.6x |
| UBA52; UBB;  RPS27A; UBC | Ubiquitin* | 40.9 | 29.7 | 2.7 | 105.3 | 54.7 | 2.4 | 2.6 | 6.0x |

STG-p62 and wt Hap1 cells were subjected to GFP-TRAP (Chromotek) purification followed by mass spectrometry. Listed are the known p62 direct interactors that resulted enriched in the STG-p62 sample compared to the wt control in two independent replicates. A Log2(LFQ)≥1 was considered as significant enrichment. Individual Log2(LFQ) values as well as the mean Log2(LFQ) (Log2(LFQ)_Avg_) are reported. The mean enrichment factor (2^Log2(LFQ)_Avg_) is also shown. LFQ: label-free quantitation.

* Full name: Ubiquitin-60S ribosomal protein L40; Ubiquitin-40S ribosomal protein S27a; Polyubiquitin-B; Polyubiquitin-C.
