# Supplementary material for: p62 filaments capture and present ubiquitinated cargos for autophagy
Source: EMBO J. 2018 Jan 17;37(5):e98308. doi: 10.15252/embj.201798308 (PMC5830917; doi:10.15252/embj.201798308)
Supplement: Supplementary file 3 — Movie EV1 [file EMBJ-37-e98308-s003.zip › README__Expanded_View_Movie_EV1.docx]

**README_ Expanded View Movie EV1**

Representative lapse of a clustering reaction conducted with mCherry-p62 and GST-GFP-4xUb. Samples with mCherry-p62 and GST-GFP-4xUb alone are also shown. Time points are indicated in each frame. The mCherry and GFP fluorescence are displayed in red and green, respectively.
