# Supplementary material for: p62 filaments capture and present ubiquitinated cargos for autophagy
Source: EMBO J. 2018 Jan 17;37(5):e98308. doi: 10.15252/embj.201798308 (PMC5830917; doi:10.15252/embj.201798308)
Supplement: Supplementary file 4 — Movie EV2 [file EMBJ-37-e98308-s004.zip › README__Expanded_View_Movie_EV2.docx]

**README_Expanded View Movie EV2**

Representative time lapse of fluorescence recovery after photobleaching experiments conducted with mCherry-p62- and GST-GFP-4xUb-containing clusters.
