# Supplementary figures and images for: p62 filaments capture and present ubiquitinated cargos for autophagy
Source: EMBO J. 2018 Jan 17;37(5):e98308. doi: 10.15252/embj.201798308 (PMC5830917; doi:10.15252/embj.201798308)

Figure 2 Source Data

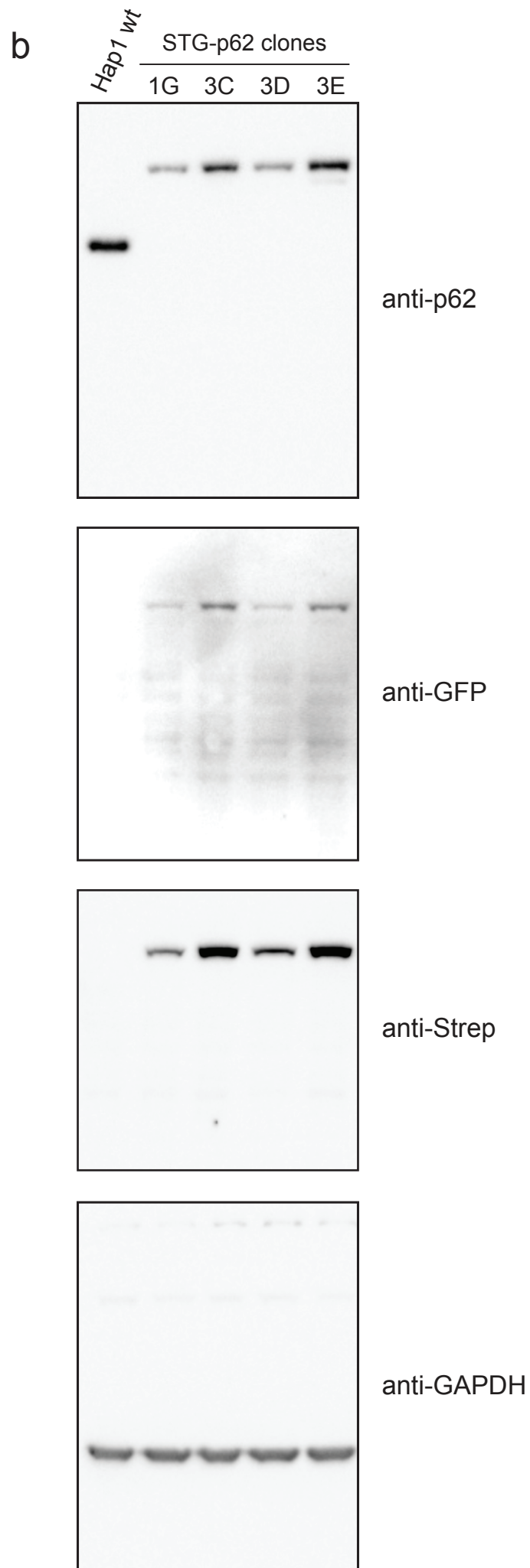

Supplement: Supplementary file 7 — Source Data for Figure 2 [file EMBJ-37-e98308-s006.pdf]

Figure 6 Source Data

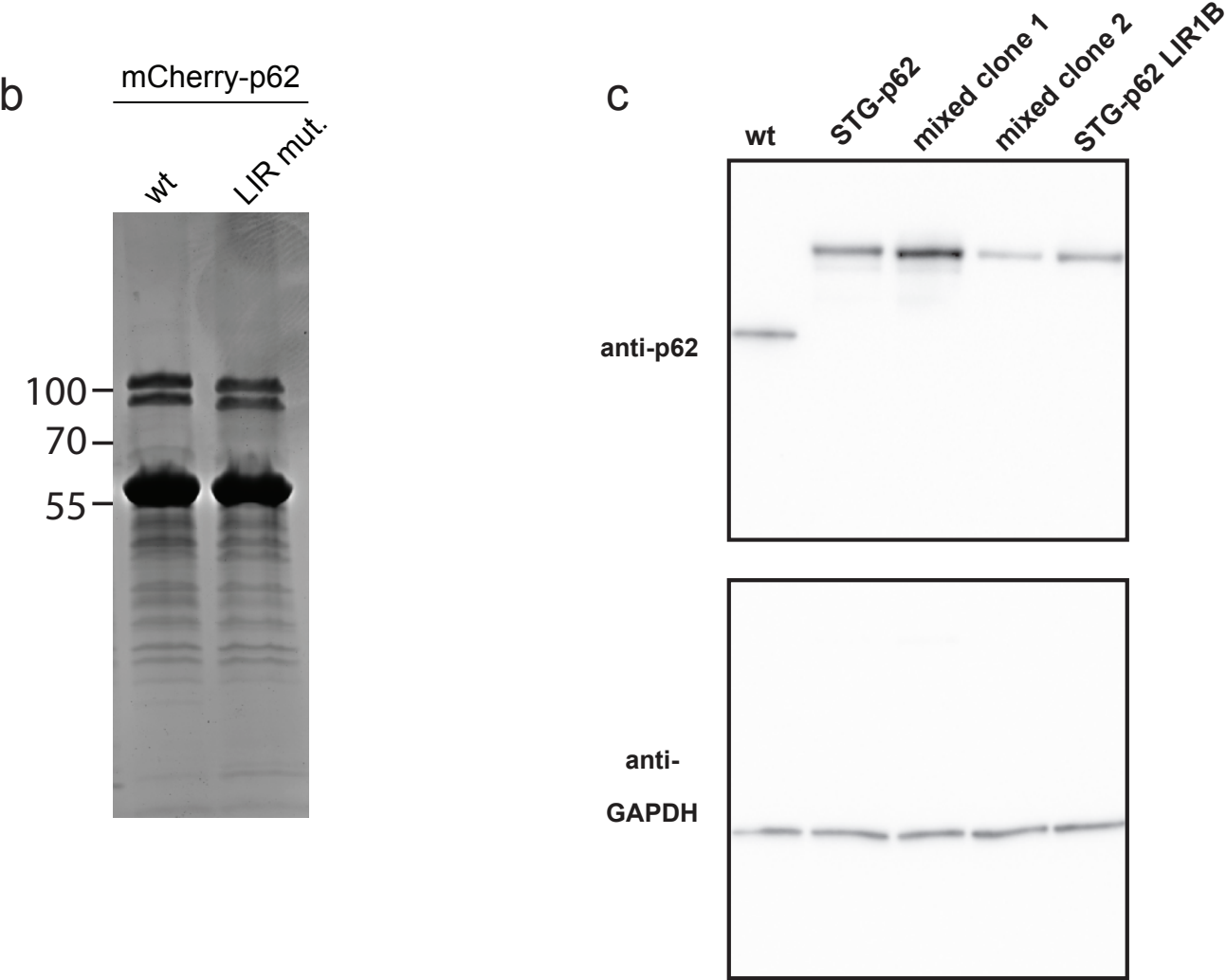

Supplement: Supplementary file 8 — Source Data for Figure 6 [file EMBJ-37-e98308-s007.pdf]
